
JOURNAL INFORMATION
==============================
NLM Title Abbreviation: Atlanta Med Surg J (1884)
NLM Journal ID: 101251623

Atlanta Medical and Surgical Journal (1884)

Publisher: Biomedical Journal Digitization Project

ARTICLE INFORMATION
==============================
PMCID: PMC9201856
PMID: 35829607
Article version: 1
Subjects: Original Communications

Retinal Surgery in Cross-eye or Strabismus

Patterson A. Bethune M.D. Atlanta, Ga.

Print publication date: 1899 Feb
Volume: 15
Volume Series: New Ser
Issue: 12
First page: 807
Last page: 810
License: This work is free of known copyright restrictions. For more information, please see PMC Back Issue Digitization.
License URL: https://creativecommons.org/publicdomain/mark/1.0/

==============================

all cases where the uterus is emptied artificially it should be done under the most strict asepsis and thoroughly drenched with bichloride or other good antiseptic hot solution.

The after-treatment consists in rest in bed and attention to such details as contribute to the comfort of the patient. Ergot may be given to aid retraction of uterus and assist involution.

---

### RETINAL SURGERY IN CROSS-EYE OR STRABISMUS.

By A. BETHUNE PATTERSON, M.D.,

ATLANTA, GA.

I deem a clearer idea of the indications in strabismus or cross-eye essential, from the fact that the impression is general, not only with the laity but the profession as well, that the cutting and setting back of a muscle to the degree of straightening, or apparently straightening, the eye is all that can be desired, from an operative standpoint.

What is to be understood by the word "cure," as applied to cross-eyes?

It is not only straightening the eye and relieving the deformity, but going a step further and restoring its normal function of seeing or operating with its fellow.

It is a fallacy to presume that this physiological function takes place as soon as the eye regains its apparent normal position. That this statement may be clearly understood, it is requisite that we first understand the conditions essential to normal or binocular vision.

In the first place, each eye receives the image of the object observed, their refractive conditions being equal. While there are two images, only one appears, caused by the exact overlapping. We speak of this phenomenon as fusion of images; but if one eye turns either in or out, however slightly, double images will manifest themselves; the greater the deviation, the greater the separation of the images. When slight, the images are nearly together; if the distance is considerable, the image of the deviating eye will not be observed unless carefully looked for. As a general thing the deviating eye quickly learns to ignore or suppress its image, a wise provision of nature, as double images are annoying and confusing.

The eye is carried in or out by the combined action of several muscles. Limited space deters me from entering into a discussion of the action of the extraneous muscles of the eyeball, individually or collectively.

In previous articles I have condemned the indiscriminate tenotomies of the internal recti muscle in convergent squint. I have pointed out the evils of the operation, and the fallacy of operating merely to improve the appearance.

I have for years contended that no surgical operation should be resorted to until the refraction was understood, all errors corrected, and atropin given a fair trial; that is, whenever the peculiar conditions call for this course of management. This treatment proved successful in a large percentage of cases, especially in the very young, when taken in hand as soon as the squint appeared.

Fifteen years or so ago, it was the rule with operators to advise a postponement of surgical interference until the squint became fixed, and the child reached a more matured age. Experience has taught us that delays in these cases are hazardous. Children should be placed under treatment as soon as the squint is observed, and every effort made to correct the deviation before resorting to tenotomy, as this operation alone is never successful in reinstating binocular vision, which is the indication I regard as by far the most important. But, on the other hand, the setting back the muscles certainly greatly complicates the case, so far as binocular vision is concerned.

Only recently the attention of a few operators has been turned to the matter of restoring to the deviating eye the faculty of working with its fellow. Heretofore the sole aim has been to straighten the eye, and thereby overcome the deformity, which, by no means, invariably proved successful.

It is essential that the horizontal and vertical meridians of the eyes be kept in their normal relations, otherwise binocular vision would be unobtainable.

It is the rule, that when the internal recti is severed, that the eye immediately rotates upon its axis, and turns upwards (hypertropia). This turning up is usually one of the complications of the convergent squint, and becomes more accentuated after tenotomy.

Mr. Nettleship used to say, when doing an operation for convergent squint: "Always tell the parent, before you operate, that you expect the eye to turn up a little. If you neglect to do so, they are apt to return and inform you of this turning." This complication renders binocular vision more difficult and tedious to obtain. This upward turning must be corrected before fusion of images can result. Eyes, after tenotomy, are never in a normal position, however much they appear to be.

How is binocular vision to be determined?

I have stated that straightening the eye does not insure physiological usefulness. Then how are we to know when the eye becomes cognizant of its image? There are two tests. One is simple and easily made thus: Take some reading-matter where the lines extend across the page, hold it at the usual reading distance, then hold a pencil or penholder vertically four or five inches in advance of the reading-matter. It will be observed that none of the letters will be obscured by the pencil, if binocular vision exists; but if only one eye is doing the work, some of the letters will be obscured, which will necessitate the removal of pencil or moving of the head to see obscured letters. If this experiment is being made by one who possesses binocular vision and desires proof of the above statement, he has only to close one eye to find a few of the letters obscured by the pencil.

Another test, the name of which has escaped my memory, consists of six letters stained on glass, alternate red and green. Now, of course, either eye would perceive the red and green letters; but place before the right eye a red glass, which does not admit the passage of green rays, and a green glass, through which red rays do not pass, before the left eye, and if the normal physiological condition is present, both red and green letters will be observed; but if only one eye is doing the work, and that be the right eye, then only the red letters will be seen, because nothing green can be seen through the red glass, and no red object can be observed through green. If only the left eye is doing the work, only the green letters will be seen. I prefer the latter test, as no errors can arise in making it. This test is made at a distance of fifteen or twenty feet. Place the colored letters in front of a window, candle or gas-jet.

I have succeeded in establishing binocular vision in only a few cases where the muscle had been set back by tenotomy. It is apropos to state that, owing to the tediousness of these cases, and the time and patience required, very few cases of this character are undertaken. These patients must go through life possessing two good eyes, normal in acuteness, and using only one.

The only way to avoid this very undesirable condition of affairs is to steer clear of tenotomies, and rely upon the operation of shortening the so-called weak muscle.

In a future article I propose to take up the operation of shortening a muscle, and the methods used in restoring binocular vision.

*507 English-American Building.*

---
